# Establishment of a Porcine Small Intestinal Organoid Model for Porcine Rotavirus Infection

**DOI:** 10.3390/ani16172670

**Published:** 2026-08-25

**Authors:** Huangsiwu Wei, Wenjie Wu, Maokang Liu, Mengchen Li, Juan Zhang, Shunzhou Deng

**Affiliations:** 1 Department of Preventive Veterinary Medicine, College of Animal Science and Technology, Jiangxi Agricultural University, Nanchang 330045, China; weihuangsiwu@126.com (H.W.); 18837053516@163.com (W.W.); liumaokang2022@163.com (M.L.);; 2 Lushan Botanical Garden, Jiangxi Province and Chinese Academy of Sciences, Lushan 332900, China

**Keywords:** porcine rotavirus, small intestinal organoid, three-dimensional culture, two-dimensional culture, inflammatory cytokines

## Abstract

Porcine rotavirus (PoRV), a major enteric pathogen, causes severe diarrhea, dehydration, high morbidity, and mortality, leading to significant economic losses in the porcine industry. In this study, we established a porcine small intestinal organoid culture system and developed an in vitro model for PoRV infection. Jejunal crypts were isolated from neonatal piglets and subsequently cultured to generate porcine small intestinal organoids in both three-dimensional (3D) and two-dimensional (2D) organoid formats, properties of which were verified via morphological assessment and immunostaining for Lgr5, villin, Ki-67, and lysozyme. Furthermore, a PoRV infection model was established in both 3D and 2D organoid cultures, and growth kinetics demonstrated efficient PoRV replication. PoRV infection also significantly increased the expression of inflammatory cytokines, including IL-6 and IFN-α. Collectively, these results demonstrate that the established porcine small intestinal organoid models support efficient PoRV replication and provide a physiologically relevant platform for studying PoRV pathogenesis and evaluating potential antiviral strategies.

## Simple Summary

Studying porcine rotavirus and developing effective interventions can be challenging without a reliable laboratory model that mimics a pig’s gut. We developed a novel culture system by growing miniature, simplified versions of the pig small intestine, called organoids, from neonatal piglet tissue. We successfully infected these organoids with porcine rotavirus and observed that the virus replicated efficiently. Infected organoids also showed an increased production of key inflammatory signals, mirroring a real infection. This new model provides a physiologically relevant tool, allowing researchers to study the virus’s disease mechanisms and test potential antiviral treatments without constantly relying on live animals.

## 1. Introduction

Porcine rotavirus (PoRV) is a major etiological agent of viral diarrhea in pigs worldwide. PoRV primarily infects neonatal piglets, causing severe watery diarrhea, vomiting, dehydration, and metabolic acidosis, which can impair growth performance, resulting in substantial economic losses in the swine industry [1,2,3]. PoRV primarily infects villous enterocytes—the mature, absorptive cells lining the small intestine [4]. Although IPEC-J2 and MA104 are widely used as in vitro models for investigating enteric pathogens, these conventional cell culture systems fail to recapitulate the complex three-dimensional (3D) architecture, cell–cell interactions, and cellular heterogeneity of the native intestinal epithelium [5,6]. In contrast, in vivo animal models are associated with high costs, limited experimental throughput, and ethical concerns [7,8]. In recent years, the development of organoid technology has provided a promising alternative to overcome these limitations [9]. By culturing adult intestinal stem cells within a 3D extracellular matrix in vitro, self-organizing structures that recapitulate key features of the intestinal crypt–villus architecture can be generated [10,11]. These organoids closely mimic the cellular composition, spatial organization, and functional characteristics of the original tissue, thereby providing a physiologically relevant platform for investigating intestinal development, host–pathogen interactions, infection mechanisms, and tissue regeneration [12].

Intestinal organoids comprise highly polarized epithelial cells that differentiate into the major lineages found in the native intestinal tissue, including mature goblet cells, enterocytes, Paneth cells, and enteroendocrine cells. Within these organoids, intestinal stem cells undergo continuous self-renewal and give rise to transit-amplifying progenitor cells, which subsequently differentiate along the crypt–villus axis into absorptive enterocytes, mucus-secreting goblet cells, and hormone-secreting enteroendocrine cells [13]. The application of intestinal organoids has substantially advanced virological research by providing physiologically relevant in vitro models of viral infection. For instance, Krüger et al. demonstrated that SARS-CoV-2 can infect multiple cell types within human intestinal organoids, resulting in ultrastructural alterations and the disruption of epithelial integrity—a finding that may contribute to the gastrointestinal manifestations of COVID-19 [14]. In the context of rotavirus infection, Saxena et al. reported pronounced luminal distension in human intestinal organoids following infection, recapitulating a characteristic feature of rotavirus-induced diarrhea. This model has provided a valuable platform for studying rotavirus biology, including host restriction, cell-type specificity, and virus-induced fluid secretion [15]. Furthermore, Finkbeiner et al. demonstrated that human intestinal organoids efficiently support the replication of multiple clinically relevant rotavirus strains, with viral infection detected in both epithelial cells and mesenchymal cell populations. These findings highlight the broad potential of intestinal organoid systems in studying viral pathogenesis [16].

Although intestinal organoid systems derived from mice and humans have been well established, the development of porcine small intestinal organoids for studying rotavirus infection [17,18,19] remains technically challenging, primarily due to the relatively low efficiency of crypt isolation and our incomplete understanding of the mechanisms governing epithelial differentiation and maturation [20,21,22,23,24,25]. Moreover, previous studies have demonstrated that both the crypt isolation procedure and the physicochemical properties of the extracellular matrix are critical determinants of organoid formation efficiency and functional maturation. To date, studies of PoRV have predominantly relied on conventional cell lines, such as MA104 cells, or animal models, neither of which fully recapitulate the intestinal epithelial architecture and host-specific microenvironment relevant to natural infection [26]. Although human intestinal organoids have provided valuable insights into rotavirus pathobiology [15,16], and recent studies have demonstrated the feasibility of porcine enteroid/organoid models, porcine small intestinal organoid culture systems for PoRV infection have not yet been comprehensively established. In particular, the optimization of inoculation strategies and characterization of host inflammatory responses remain insufficiently investigated.

Accordingly, this study established a stable and efficient porcine small intestinal organoid culture system and systematically characterized its stem cell activity, epithelial differentiation, and functional maturation. We further developed an in vitro PoRV infection model, compared viral replication efficiency using different inoculation strategies, and evaluated virus-induced cytopathic effects and inflammatory cytokine responses. Collectively, these findings establish a robust and physiologically relevant platform for investigating the pathogenesis of porcine enteric viruses, elucidating cell–host interactions. Furthermore, this organoid-based system provides a valuable foundation for the preclinical evaluation of antiviral interventions.

## 2. Materials and Methods

### 2.1. Virus Strain, Reagents and Ethics Statement

The PoRV-JJ strain was originally isolated from diarrheic fecal samples collected from diseased piglets on a pig farm in Jiujiang City, Jiangxi Province, China. Prior to use in the present study, the virus was propagated in MA104 cells for 8 passages, and the viral stock at passage 13 (P13) was used for all infection experiments. The GenBank accession numbers for the PoRV-JJ strain are PZ775684–PZ775694. A clinically healthy neonatal piglet, aged one day, was used as a donor for jejunal crypt isolation and organoid culture. Jejunal tissues were collected immediately after humane euthanasia performed by exsanguination under anesthesia, in accordance with the approved animal ethics protocol. Before inclusion in the study, the donor piglet was confirmed to be negative for common enteric pathogens, including transmissible gastroenteritis virus (TGEV), porcine epidemic diarrhea virus (PEDV), and porcine rotavirus (PoRV), by RT-PCR. All animal experimental procedures and animal care protocols were approved by and performed in accordance with the Guidelines for the Care and Use of Laboratory Animals of Jiangxi Agricultural University (JXAULL-2026.05-11). A mouse monoclonal antibody against PoRV VP6 was generated and stored in our laboratory.

### 2.2. Isolation of Porcine Small Intestinal Crypts and Culture and Passaging of Three-Dimensional Organoids

Small intestinal crypts were isolated from the jejunum of neonatal piglets. Briefly, approximately 8 cm of proximal jejunum was collected, and the surrounding adipose tissue and mesentery were carefully removed. The villus epithelium was then gently scraped away, and the remaining tissue was thoroughly rinsed with PBS and cut into approximately 2 × 2 mm fragments. The tissue fragments were incubated in 5 mL of ice-cold 0.5 mM EDTA solution with gentle shaking at room temperature for 30 min. Following incubation, the EDTA solution was replaced with PBS-supplemented 10% fetal bovine serum (FBS). Intestinal crypts were released by vigorous pipetting, and the resulting suspension was sequentially filtered through 150-mesh and 100-mesh cell strainers. The filtrate was collected and centrifuged at 300× *g* for 5 min at 4 °C. The resulting pellets containing intestinal crypts were resuspended in PBS and manually assessed using a hemocytometer with trypan blue staining.

For organoid culture, isolated crypts were resuspended in Matrigel (Corning, New York, NY, USA, Cat#356231), and 50 μL droplets containing approximately 500 crypts were seeded into individual wells of a 24-well plate. After polymerization of the Matrigel at 37 °C, 800 μL of IntestiCult™ Organoid Growth Medium (Human) (STEMCELL Technologies, Vancouver, BC, Canada; Cat# 06010) was added to each well. Organoid growth and morphology change was monitored under microscope observation, and the culture medium was replaced each 2–3 days. After 5–7 days of culture, organoids were harvested by pipetting with Dulbecco’s Phosphate-Buffered Saline (DPBS) and dissociated with Gentle Cell Dissociation Reagent (STEMCELL Technologies, Vancouver, BC, Canada Cat# 100-0485). The dissociated organoids were subsequently resuspended in fresh Matrigel and passaged for further expansion.

### 2.3. Cryopreservation and Resuscitation of Porcine Small Intestinal Organoids

Well-developed organoids were harvested by gentle pipetting and resuspended in CryoStor^®^ CS10 Cell Freezing Medium (STEMCELL Technologies, Vancouver, BC, Canada; Cat. No. 100-1061). The organoid suspension was aliquoted into cryovials, which were placed in a BeyoCool™ Cell Freezing Container (Beyotime, Shanghai, China) and cooled at −80 °C overnight before being transferred to liquid nitrogen for long-term storage. For organoid recovery, cryovials were rapidly retrieved from liquid nitrogen and immediately thawed in a 37 °C water bath with gentle agitation until completely thawed. The thawed organoid suspension was transferred to a centrifuge tube containing DPBS and centrifuged at 300× *g* for 5 min at 4 °C. This washing step was repeated to remove residual cryopreservation medium. The recovered organoids were then resuspended in organoid growth medium and returned to culture for further expansion.

### 2.4. Culture of Porcine Small Intestinal Two-Dimensional Organoids

Based on the established three-dimensional (3D) porcine small intestinal organoid culture system, organoids were dissociated into small cell clusters by incubation with Gentle Cell Dissociation Reagent (STEMCELL Technologies, Vancouver, BC, Canada) at 37 °C for 35 min. For two-dimensional (2D) culture, each well of a 24-well plate was precoated with 300 μL of high-glucose DMEM supplemented with 1.5% Matrigel (Corning, New York, NY, USA) and incubated at 37 °C for 1 h to allow the coating matrix to polymerize. The dissociated organoid fragments were then seeded into the precoated wells and maintained in organoid growth medium. Following attachment, the cells progressively proliferated and formed a confluent monolayer within approximately 24 h.

### 2.5. Establishment of a Porcine Rotavirus Infection Model in Porcine Small Intestinal Organoids

To establish a PoRV infection model in porcine small intestinal organoids, three viral inoculation strategies were evaluated to determine the optimal infection approach. For all infection experiments, the PoRV-JJ viral stock (passage 13, titer: 6.5 × 10^6^ PFU/mL) was pre-activated with 15 μg/mL trypsin at 37 °C for 1.5 h prior to inoculation. An equivalent viral dose (MOI ≈ 1.0) was used for all infection routes to enable direct comparison of infection efficiency. Three infection routes were tested as follows: Route 1: Fully differentiated 3D organoids were harvested from Matrigel and mechanically disrupted into small fragments prior to viral adsorption, allowing increased exposure of the luminal epithelial surface. The organoid fragments were incubated with trypsin-activated PoRV at 37 °C for 3 h. Following viral adsorption, the organoids fragments were resuspended in organoid growth medium supplemented with 10 μM Y-27632 and seeded into 24-well plates pre-coated with Matrigel for subsequent culture. Route 2: Activated PoRV was directly added to wells containing fully differentiated organoids maintained within Matrigel. Following incubation at 37 °C for 3 h, the viral inoculum was removed, and organoid growth medium supplemented with 10 μM Y-27632 was added for continued culture. Because the organoids remained intact and embedded in Matrigel, viral access to the luminal apical surface was expected to be relatively restricted, with exposure occurring primarily at the externally accessible epithelial. Route 3: Fully differentiated organoids were released from Matrigel and incubated with trypsin-activated PoRV in suspension at 37 °C for 3 h, with gentle agitation every 30 min. Unlike Route 1, the organoids were maintained intact without mechanical disruption. Following viral adsorption, the organoid–virus suspension was resuspended in fresh Matrigel, seeded into 24-well plates, and maintained in organoid growth medium supplemented with 10 μM Y-27632. The three inoculation strategies were subsequently compared based on viral replication efficiency and infection-associated responses to identify the most suitable approach for establishing the PoRV infection model.

For infection of 2D organoid cultures, confluent cell monolayers were washed three times with DMEM and then incubated with the trypsin-activated virus at an MOI of 1. The cells were incubated at 37 °C for 3 h to allow for viral adsorption. Following incubation, the inoculum was removed, and the cells were maintained in organoid growth medium.

Mock-infected controls were processed in parallel following identical procedures as the infected groups, except that an equal volume of mock inoculum, consisting of trypsin-treated supernatant from uninfected cells, was used in place of PoRV. For all infection groups, culture supernatants were collected at 1, 36, and 72 h post-infection (hpi) for viral load analysis.

### 2.6. Indirect Immunofluorescence Assay (IFA) for Detection of Intestinal Organoid Markers

For three-dimensional (3D) organoids, well-developed organoids were collected, fixed with 4% paraformaldehyde, embedded in paraffin, and sectioned. The sections were deparaffinized in xylene and rehydrated through a graded ethanol series, followed by antigen retrieval in citrate buffer (pH 6.0) using microwave heating. After cooling down to room temperature, the sections were blocked with 3% bovine serum albumin (BSA) for 2 h. For two-dimensional (2D) organoids, samples were fixed directly in 24-well plates with 4% paraformaldehyde, permeabilized with 1% Triton X-100 for 25 min, and blocked with 3% BSA for 2 h. Both 3D and 2D organoid samples were subsequently incubated overnight at 4 °C with the following primary antibodies (all diluted 1:100): anti-LGR5 polyclonal antibody (Proteintech Group, Inc., Wuhan, China, Cat# 30007-1-AP), anti-villin polyclonal antibody (Proteintech Group, Inc., Wuhan, China, Cat# 16488-1-AP), anti-Ki-67 polyclonal antibody (Proteintech Group, Inc., Wuhan, China, Cat# 28074-1-AP,), and anti-lysozyme polyclonal antibody (Proteintech Group, Inc., Wuhan, China, Cat# 150113-1-AP). Following three washes with PBS, the samples were incubated with FITC-conjugated goat anti-rabbit IgG (H+L) cross-adsorbed secondary antibody (Proteintech Group, Inc., Wuhan, China; Cat# B900210, dilution: 1:500) for 12 h at 4 °C. The nuclei were counterstained with DAPI for 10 min. After washing, the samples were mounted and examined using a confocal microscope (Nikon, Tokyo, Japan; ECLIPSE C1).

### 2.7. Hematoxylin and Eosin (HE) Staining of Porcine Small Intestinal Three-Dimensional Organoid Sections

3D organoids were collected, fixed with 4% paraformaldehyde, embedded in paraffin, and sectioned. The sections were deparaffinized in xylene and rehydrated through a graded ethanol series. Subsequently, the sections were stained with hematoxylin for 3–8 min, rinsed thoroughly with water, and differentiated in 1% acid alcohol for several seconds. The sections were then blued under running water and counterstained with eosin for 1–3 min. Following staining, the sections were rinsed with tap water, dehydrated through a graded ethanol series, cleared in xylene, and mounted with neutral balsam. The stained sections were subjected to light microscopic observation.

### 2.8. Detection of Viral Copies by Quantitative Real-Time PCR (qRT-PCR)

Culture supernatants from PoRV-infected organoids were collected at the indicated time points for viral copy determination, as well as those from mock-infected cells. Total RNA was extracted using RNAiso reagent (TaKaRa, Kusatsu, Japan; Cat# 9108). Viral RNA levels were quantified by qRT-PCR using primers and a TaqMan probe targeting the PoRV NSP3 gene. The primers were as follows: forward primer, 5′-ACCATCTWCACRTRACCCTC-3′; reverse primer, 5′-GGTCACATAACGCCCCTATA-3′; and the TaqMan probe is 5′-FAM-ATGAGCACAATAGTTAAAAGCTAACACTGTCAA-TAMRA-3′. The expected amplicon size is 87 bp. qRT-PCR was performed using the StarScript II Probe One-Step qRT-PCR Kit (GenStar, Beijing, China; Cat# A325) according to the manufacturer’s instructions.

### 2.9. Detection of Viral Protein by Indirect Immunofluorescence Assay

Three-dimensional (3D) or two-dimensional (2D) organoids were collected at 36 hpi and fixed with 4% paraformaldehyde for 20 min at 4 °C. The samples were then incubated with mouse anti-PoRV-VP6 monoclonal antibody (house-generated; dilution: 1:500) overnight at 4 °C. After washing, the samples were incubated with fluorescein (FITC)-conjugated goat anti-mouse IgG (H+L) secondary antibody (Proteintech Group, Inc., Wuhan, China, Cat# SA00003-1; dilution: 1:500) for 12 h at 4 °C. Following nuclear counterstaining with DAPI, fluorescence images were acquired using a fluorescence microscope.

### 2.10. Detection of Inflammatory Cytokines by Enzyme-Linked Immunosorbent Assay (ELISA)

The concentrations of TNF-α, IFN-α, and IL-6 in culture supernatants were measured using porcine ELISA kits according to the manufacturers’ instructions. The following kits were used: Porcine Tumor Necrosis Factor-α (TNF-α) ELISA Kit (Bioswamp, Wuhan, China; Cat# PR40133), Porcine Interleukin-6 (IL-6) ELISA Kit (Bioswamp, Wuhan, China; Cat# PR40121), and Porcine Interferon-α (IFN-α) ELISA Kit (Bioswamp, Wuhan, China; Cat# SPR40142). Briefly, culture supernatants collected from 3D organoids infected with PoRV-JJ at 0, 36, and 72 hpi were added to the ELISA plates along with the corresponding standards at concentrations ranging from 7.5 to 600 pg/mL. All samples and standards were analyzed in duplicate. The assays were performed according to the manufacturer’s protocol, and absorbance was measured at 450 nm using a microplate reader (Thermo Fisher Scientific, Waltham, MA, USA; Multiskan FC,). Cytokine concentrations were calculated based on standard curves generated from the corresponding standards.

### 2.11. Statistical Analysis

All experiments were performed with three independent biological replicates, and data are expressed as the mean ± standard deviation (SD). Statistical analyses were performed using GraphPad Prism 8. Viral replication kinetics, including Ct values across different time points and infection routes, were analyzed by two-way analysis of variance (ANOVA) followed by Tukey’s post hoc test. Cytokine concentrations between mock-infected and PoRV-infected organoids at each time point were compared using an unpaired two-tailed Student’s *t*-test. A *p* value < 0.05 was considered statistically significant. In the Section 3, exact *p* values are provided where applicable, with * *p* < 0.05, ** *p* < 0.01, and *** *p* < 0.001.

## 3. Results

### 3.1. Establishment and Characterization of Porcine Small Intestinal Organoid Culture Systems

**Figure 1:**
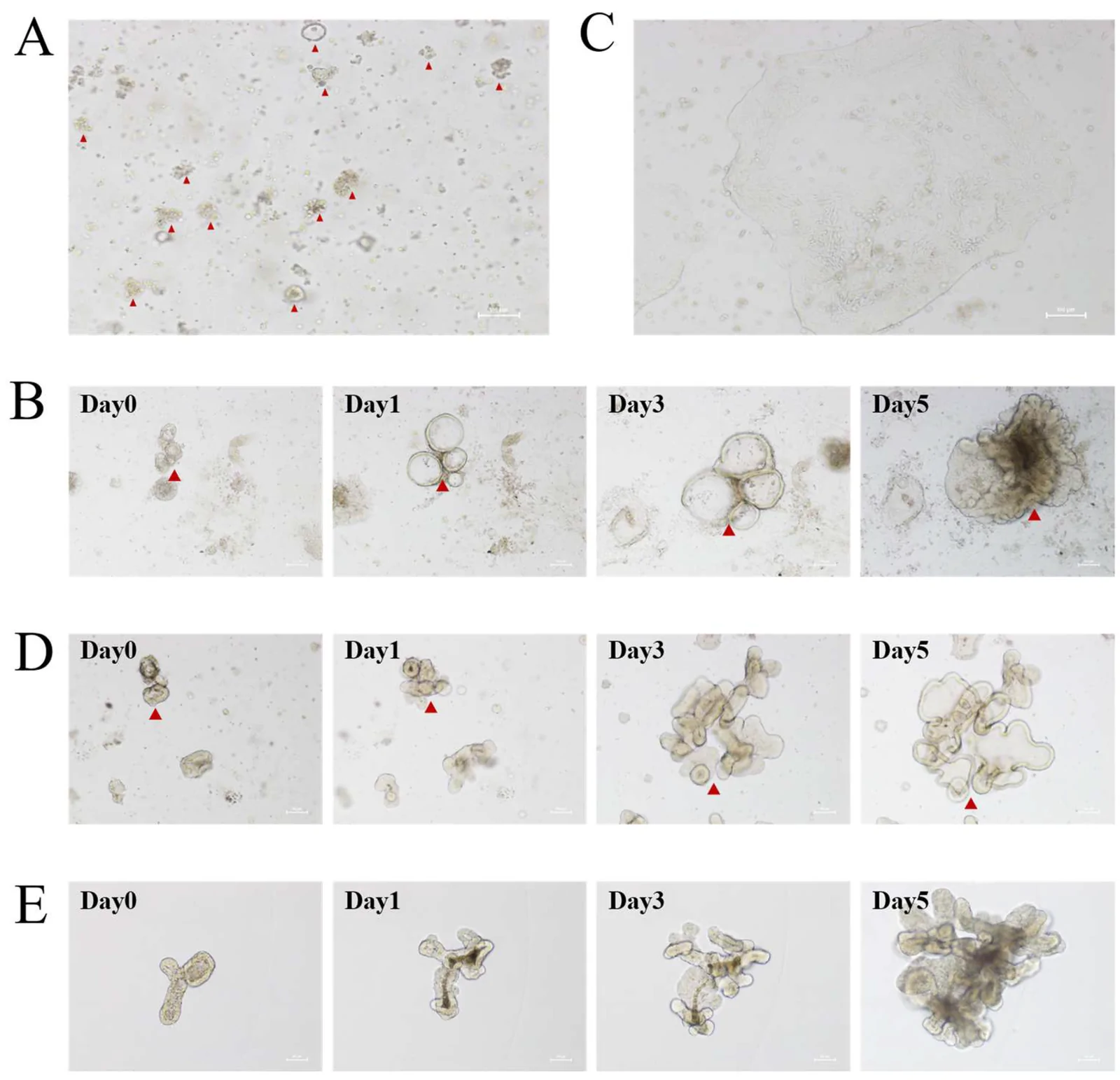
Establishment and characterization of porcine small intestinal organoids. (**A**) Crypts isolated from porcine jejunum after filtration through a 150-mesh sieve. Crypt fragments were observed under light microscopy. Scale bar: 100 μm. (**B**–**D**) Morphological observation of porcine small intestinal organoids at 0, 1, 3, and 5 days after seeding (left to right). Porcine small intestinal organoids were generated by proliferation and differentiation from isolated crypts (**B**), after five passages (**C**), or following recovery from cryopreservation (**D**). Scale bar: 100 μm. (**E**) Two-dimensional monolayer culture of intestinal epithelial cells at day 3 after seeding. Scale bar: 100 μm. Red arrows indicate crypts (**A**) and organoids (**B**,**D**).

Crypts isolated from porcine jejunum were clean, structurally intact, and evenly distributed following filtration (Figure 1A). When cultured in three dimensions, the isolated crypts underwent active proliferation and differentiation, forming hollow spheroidal organoids with central lumens by day 3. As culture progressed, the organoids gradually increased in size and developed smooth surfaces with translucent lumens, indicating sustained growth (Figure 1B).

The porcine small intestinal organoids were successfully passaged and maintained a stable three-dimensional crypt–villus-like architecture across successive passages. At passage 5, the organoids retained characteristic tubular, intestine-like morphology, with no obvious changes in overall size or surface appearance compared with passage 1. These findings indicate that the organoids exhibited high morphological stability and preserved the growth and differentiation capacity during passaging organoids (Figure 1C).

To assess the stability of cryopreserved organoids, fully developed organoids stored at −80 °C were recovered. There were no obvious morphological differences between recovered and routinely cultured organoids, indicating that freeze–thaw preservation did not markedly affect organoid viability or growth potential (Figure 1D). After reaching full expansion and differentiation, the 3D organoids were further dissociated and established as a 2D monolayer culture. The resulting cells predominantly exhibited a spindle-shaped morphology characteristic of intestinal epithelial cells, demonstrating the feasibility of establishing a 2D organoid culture system from porcine intestinal organoids (Figure 1E).

### 3.2. Characterization of Porcine Small Intestinal 3D and 2D Organoids

**Figure 2:**
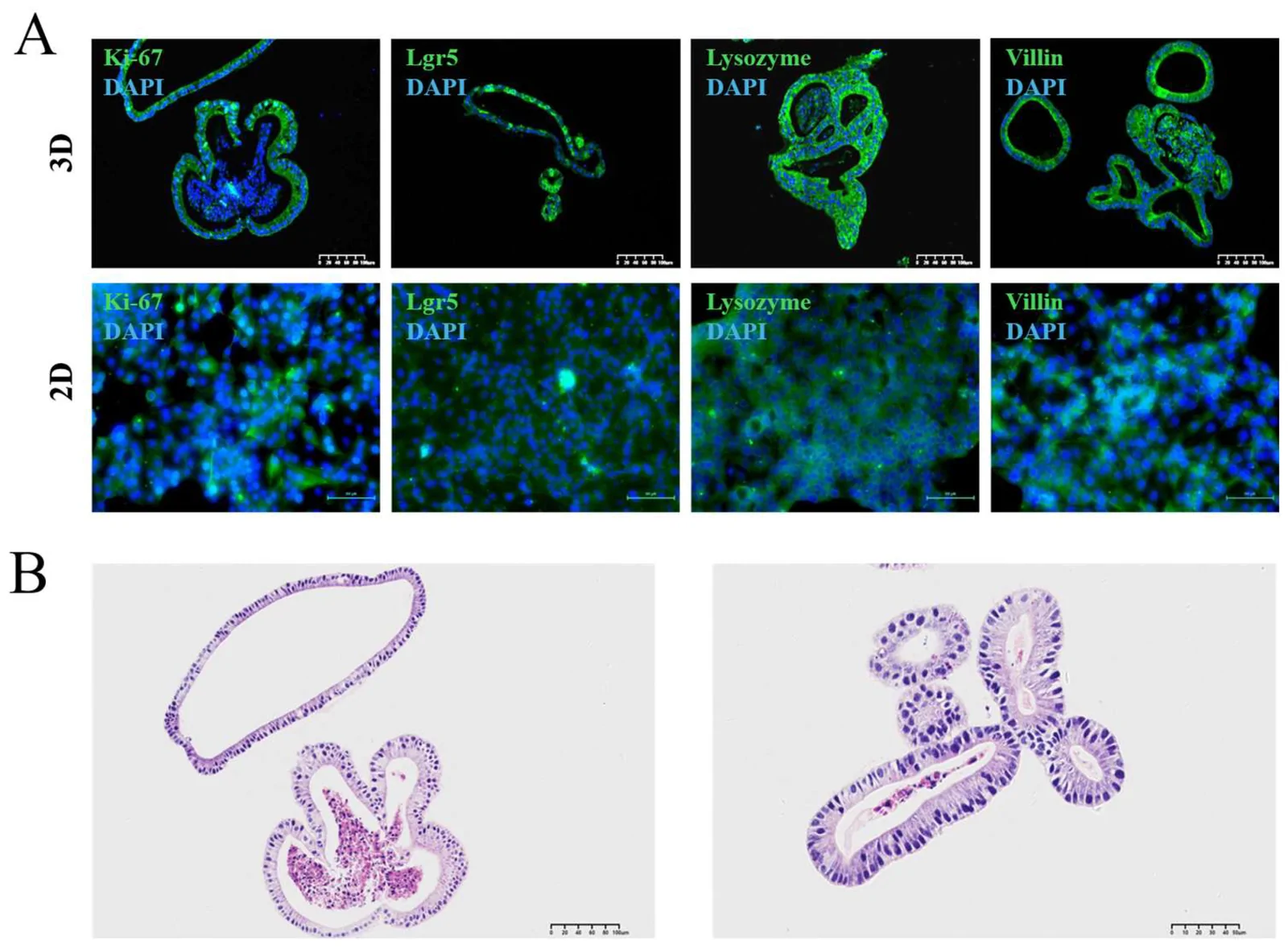
Characterization of cell differentiation in porcine small intestinal three-dimensional and two-dimensional organoids. (**A**) Immunofluorescence analysis of lineage-specific markers in 3D and 2D organoids. Organoids were stained with antibodies against Lgr5 (intestinal stem cells), villin (enterocytes), Ki-67 (proliferating cells), and lysozyme (Paneth cells). Nuclei were counterstained with DAPI. Images were acquired using a Nikon Eclipse C1 confocal microscope. Scale bar: 100 μm. (**B**) Hematoxylin and eosin (H&E) staining of 3D organoids. Scale bars: 100 μm and 50 μm.

To characterize the major intestinal epithelial cell lineages in both 3D and 2D porcine small intestinal organoids, IFA was performed using antibodies against Ki-67, Lgr5, lysozyme, and villin. As shown in Figure 2A, the 3D organoids exhibited distinct spatial distribution of intestinal epithelial cell populations. Ki-67-positive proliferating cells were periodically localized within the crypt regions, while Lgr5-positive intestinal stem cells were concentrated at the crypt base. Lysozyme-positive Paneth cells were also localized at the crypt base and exhibited characteristic granular structures. Villin was strongly polarized along the luminal surface, consistent with its localization to the apical microvillar membrane. In contrast, cells in the 2D monolayer culture did not exhibit an organized spatial distribution but still stained positive for Lgr5, villin, Ki-67, and lysozyme, indicating that multiple intestinal epithelial cell populations were maintained in the monolayer system. H&E staining further revealed that the 3D organoids consisted of densely arranged simple columnar epithelial cells with prominent nucleoli and peripherally distributed chromatin. The overall neonatal histological morphology closely resembled that of the small intestinal epithelium of neonatal piglets (Figure 2B), further supporting the structural fidelity of the established porcine small intestinal organoid model.

### 3.3. Infection of Porcine Small Intestinal Organoids with Porcine Rotavirus

**Figure 3:**
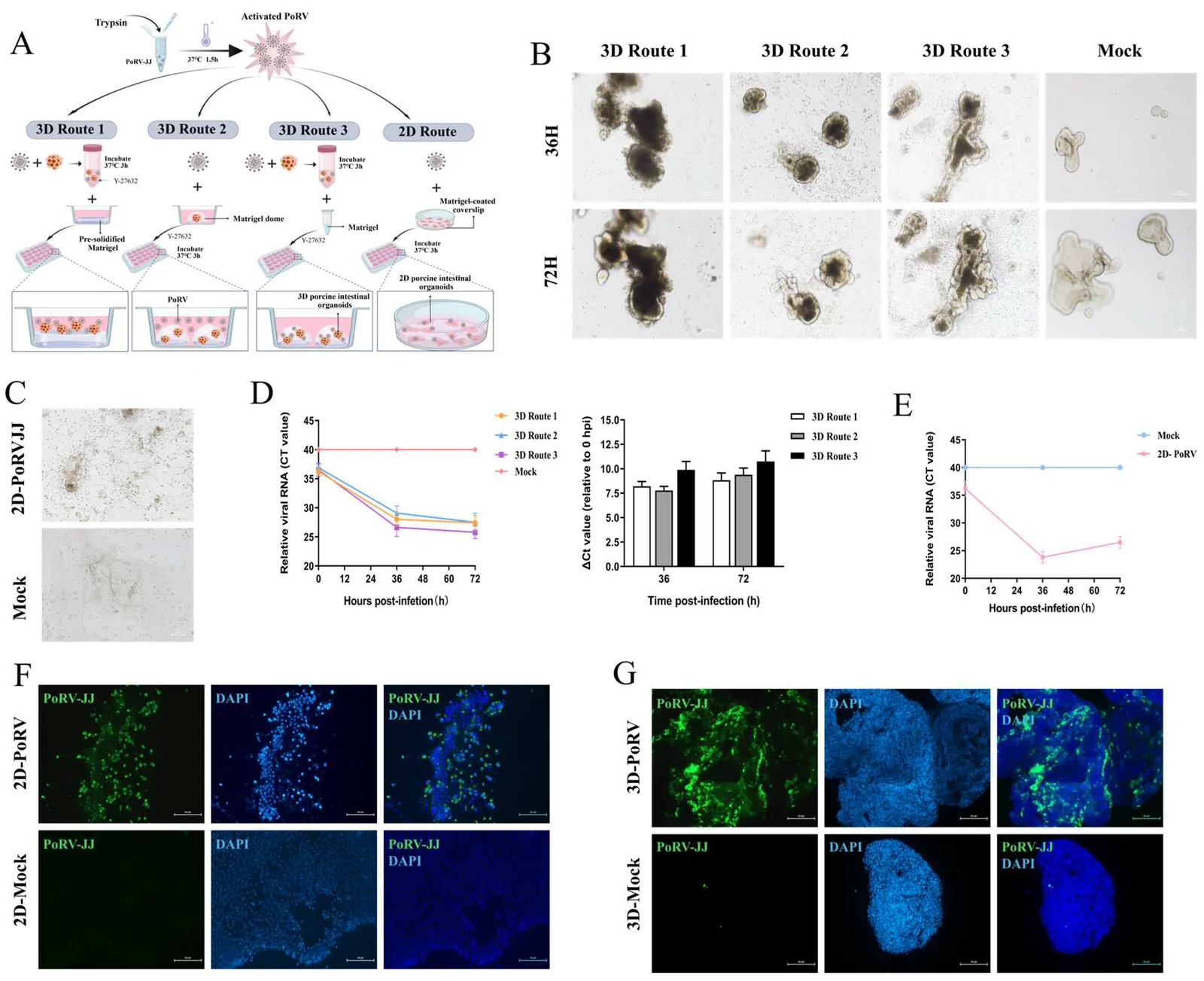
Infection of porcine small intestinal organoids with PoRV. (**A**) Schematic illustration of the three PoRV infection routes. (**B**) Representative morphological changes in 3D organoids infected with PoRV via the three infection routes at 36 and 72 hpi. Scale bar: 100 μm. (**C**) Representative morphological changes in 2D organoid-derived monolayers infected with PoRV at 72 hpi. Scale bar: 100 μm. (**D**) Replication kinetics of PoRV in 3D organoids infected via the three routes, as determined by qRT-PCR. (**E**) Replication kinetics of PoRV in 2D organoids. qRT-PCR were performed at 0, 36, and 72 hpi. Data are presented as the mean ± SD from three independent experiments. (**F**) Immunofluorescence detection of VP6 protein in 3D organoids infected with PoRV. Scale bar: 100 μm. (**G**) Immunofluorescence detection of VP6 protein in 2D organoid-derived monolayers infected with PoRV at 72 hpi. Scale bar: 100 μm.

To determine the most efficient route for PoRV-JJ in porcine small intestinal organoids, three inoculation strategies were evaluated (Figure 3A). In route 1, organoids were incubated with PoRV and then overlaid and seeded into pre-coated Matrigel. In route 2, activated PRoV was directly added to wells containing fully differentiated organoids for vial adsorption. In route 3, PoRV was mixed with organoids before the mixture was reseeded in Matrigel for further culture.

In 3D organoids, all three inoculation routes induced marked cell shedding and organoid swelling at 36 hpi, which became more pronounced at 72 hpi and was accompanied by increased cell death. Among the three routes, mixing PoRV with organoids before reseeding in Matrigel produced the most pronounced cytopathic effects and was therefore selected for subsequent experiments. In 2D organoid-derived monolayers, PoRV infection similarly induced extensive cell shedding and widening of intercellular spaces at 36 hpi (Figure 3B,C).

qRT-PCR analysis confirmed robust viral replication in both culture systems. In 3D organoids, all three inoculation routes supported rapid replication within 36 h, with route 3 showing the highest replication efficiency, as indicated by a decrease in Ct values from 37.57 at baseline to 27.38 at 36 hpi and 25.79 at 72 hpi (Figure 3D). In 2D organoid-derived monolayers, Ct values decreased from 36.17 to 23.81 at 36 hpi and then subsequently increased slightly at 72 hpi, indicating that viral replication peaked at 36 hpi (Figure 3E).

IFA further confirmed productive PoRV infection, as evidenced by strong VP6 fluorescence signals in both infected 3D and 2D organoids, whereas no detectable VP6 signal was observed in mock-infected organoids (Figure 3F,G).

### 3.4. Monitoring of Inflammatory Cytokines in Porcine Small Intestinal Three-Dimensional Organoids

**Figure 4:**
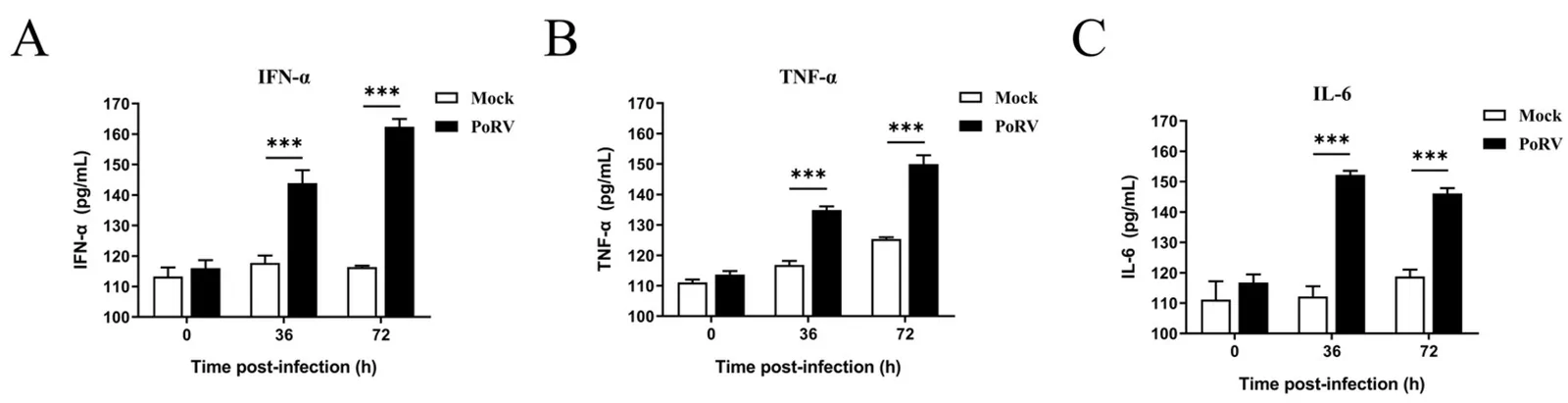
Dynamic changes in inflammatory cytokine in porcine small intestinal 3D organoids during PoRV infection: (**A**) IFN-α, (**B**) TNF-α, and (**C**) IL-6 concentrations in culture supernatants of porcine small intestinal organoids infected with PoRV at 0, 36, and 72 hpi, as determined by ELISA. Data are presented as mean ± SD (n = 3). *** *p* < 0.001.

To evaluate the inflammatory response of porcine small intestinal organoids following PoRV infection, the concentrations of interferon-alpha (IFN-α), tumor necrosis factor-alpha (TNF-α), and interleukin-6 (IL-6) in culture supernatants were measured by ELISA at 0, 36, and 72 hpi. The concentrations of IFN-α (Figure 4A), TNF-α (Figure 4B), and IL-6 (Figure 4C) were significantly upregulated at both 36 hpi and 72 hpi compared with those at 0 hpi. There findings indicate that PoRV infection induces a robust inflammatory response in porcine small intestinal organoids, further supporting the suitability of this model for recapitulating PoRV-induced intestinal inflammation.

## 4. Discussion

This study established a stably passaged porcine small intestinal organoid system as an in vitro model for investigating swine intestinal biology and PoRV infection. The organoids maintained relatively consistent growth and morphology during serial passaging and after freeze–thaw recovery, indicating their potential suitability for long-term experimental applications. Histological analysis showed an organoid architecture resembling the crypt-like organization of the small intestinal epithelium. In addition, the expression of Lgr5, Ki-67, villin, and lysozyme indicated the presence of stem/progenitor, proliferative, absorptive, and Paneth cell populations, respectively. These findings are consistent with previous studies showing that intestinal stem cells can generate self-organizing crypt–villus-like structures in vitro [27] and with reports describing the establishment and application of intestinal organoid systems in different animal species [28,29,30,31]. Collectively, the present results suggest that the established porcine organoids retain several key epithelial lineages and differentiation characteristics. However, because only selected epithelial markers were examined, further functional assays will be required to fully assess the physiological fidelity of this model.

The efficiency and viability of crypt isolation are important factors influencing organoid establishment and reproducibility [11,32]. In the present study, EDTA-based treatment and filtration through a 150-mesh sieve were used during crypt isolation. These procedures may have helped preserve crypt structures and remove larger tissue debris. However, because the individual effects of EDTA treatment and filtration were not evaluated in separate comparative experiments, their specific contributions to organoid viability and formation efficiency cannot be determined from the present data. Further optimization studies directly comparing different isolation conditions would be useful for clarifying these effects. More generally, optimization of organoid culture conditions remains important for improving the reproducibility and physiological relevance of organoid-based models [32,33].

We also compared three inoculation strategies for establishing the PoRV–organoid infection model. Route 1, involving mechanical disruption of organoids before viral adsorption and subsequent reseeding, produced the highest PoRV yield under the conditions tested. Mechanical disruption may have increased the exposed epithelial surface and improved viral access to susceptible cells, whereas the intact organoids used in Routes 2 and 3 may have limited access to the luminal epithelial surface. Because receptor localization, epithelial polarity, and the extent of epithelial disruption were not evaluated, the mechanisms underlying these differences remain uncertain. Further studies using receptor and polarity markers, as well as controlled apical and basolateral inoculation, are warranted. The observed cell shedding and cellular swelling were consistent with cytopathic changes reported in rotavirus-infected human intestinal enteroids [15,16]. The 2D cultures in the present study were derived from dissociated organoids and maintained as epithelial monolayers; therefore, they should be regarded as organoid-derived 2D cultures rather than conventional cell-line models. Differences between the 3D and organoid-derived 2D systems may reflect variations in epithelial architecture, surface accessibility, polarity, and differentiation state [17,25]. These systems should consequently be considered complementary models for studying PoRV infection [34,35,36]. Organoid models have been increasingly used in disease modeling, therapeutic development, and regenerative medicine [37,38]. In addition, organoid-derived extracellular vesicles have recently attracted attention as potential mediators of intercellular communication and as biomedical tools [39]. These broader applications highlight the versatility of organoid-based systems. In the context of enteric viral infection, organoids may provide a controllable epithelial model for examining virus-induced cellular responses and for evaluating candidate antiviral interventions. However, the organoids used in the present study primarily represented the intestinal epithelial compartment and did not fully reproduce the contributions of immune cells, stromal cells, vascular components, or systemic inflammatory responses. Therefore, their ability to model inflammation and tissue repair remains limited. Despite these limitations, previous comparative studies have demonstrated that organoid-based models can recapitulate key aspects of the in vivo immune response to rotavirus infection, including cytokine induction profiles and epithelial transcriptional changes. Nevertheless, systematic comparisons between organoid-derived and in vivo immune responses remain limited, particularly for porcine rotavirus, and represent an important area for future investigation.

In the present study, PoRV infection was associated with changes in inflammatory mediators in the organoid model. These findings are consistent with previous proteomic analyses showing that PoRV infection can alter inflammatory and host-response pathways in porcine intestinal epithelial cells [40]. However, the mechanisms underlying these inflammatory changes were not directly investigated in the current study. In particular, no experiments were performed to determine whether specific signaling pathways, such as TLR2/NF-κB, were responsible for the observed response. Therefore, these pathways should not be presented as established mechanisms based solely on the current data. Future studies should examine pathway activation using appropriate molecular markers or pharmacological and genetic inhibition approaches.

The contribution of immune cells to PoRV-induced intestinal inflammation and repair also warrants further investigation. Recent work using macrophage-augmented intestinal organoids demonstrated that incorporating immune cells can improve the investigation of virus–host interactions and therapeutic responses [41]. Such co-culture models may be particularly useful for examining the interaction between epithelial infection, innate immune activation, and tissue repair. Thus, future studies incorporating macrophages or other immune cell populations may help determine whether the inflammatory response observed in the present epithelial organoid system is modified by immune–epithelial interactions.

## 5. Conclusions

In summary, we established a stable, serially passaged porcine small intestinal organoid system that retained key intestinal epithelial lineages and crypt-like structural organization. Both 3D and 2D culture formats supported PoRV replication under the conditions tested. Among the three inoculation strategies evaluated, reseeding a virus–organoid mixture resulted in the highest PoRV production in 3D organoids. PoRV infection was also associated with changes in inflammatory mediators in the organoid model. Therefore, this porcine organoid-based infection model provides a promising platform for investigating PoRV replication, host–pathogen interactions, and antiviral interventions. Further validation using porcine intestinal tissues, animal models, and organoid co-culture systems will be important to determine the physiological relevance and broader applicability of this model.

## Data Availability

The raw data supporting the conclusions of this article will be made available by the authors on request.
